# Icing and Anti-Icing Performance of Superhydrophobic-Coated Steel Members in Long-Span Transmission Towers

**DOI:** 10.3390/ma19153224

**Published:** 2026-07-29

**Authors:** Shijun Huang, Lang Wang, Mengqi Li, Jiao Zhu, Chengyu Wang, Ruoqiang Feng

**Affiliations:** 1 Construction Branch, State Grid Jiangsu Electric Power Co., Ltd., Nanjing 210000, China; huangshijun@js.sgcc.com.cn (S.H.); zhujiao90@163.com (J.Z.); 13914710577@163.com (C.W.); 2 School of Civil Engineering, Southeast University, Nanjing 211189, China; 220241564@seu.edu.cn (M.L.); hitfeng@163.com (R.F.); 3 The Key Laboratory of Concrete and Prestressed Concrete Structures of the Ministry of Education, Southeast University, Nanjing 211189, China

**Keywords:** long-span transmission tower, superhydrophobic materials, steel member, icing characteristics, ice reduction rate

## Abstract

Long-span transmission towers in the Yangtze River basin are exposed to coupled low-temperature, high-humidity and strong-wind conditions, which promote nonuniform ice accretion on steel members and increase structural loads and ice-shedding risks. Although superhydrophobic coatings are promising passive anti-icing materials for civil infrastructure, most existing evaluations use idealized flat or cylindrical specimens and do not capture the geometry, substrate condition and coating uniformity of in-service tower members. Here, a multifactor coupled icing simulation system was developed, and comparative icing tests were conducted on three representative steel members (aged plain circular steel tube, new galvanized circular steel tube and new galvanized angle steel) under controlled temperature, wind speed, spray rate and icing duration. For uncoated members, ice mass increased with supercooling degree and spray rate, first increased and then decreased with wind speed, and exhibited a decelerating growth pattern within 24 h. The superhydrophobic coating reduced ice mass, ice thickness and circumferential nonuniformity under all tested conditions, but its effectiveness depended strongly on environmental loading and member geometry. Under reference conditions, the ice-reduction rates reached 41%, 45% and 38% for the three members, respectively, and remained 27–32% after 24 h of icing. Smooth circular substrates showed the best coating response, whereas angle steel was less effective because edge-induced flow distortion and poor coating uniformity promoted local wetting failure. Performance degradation under harsh conditions was associated with accelerated freezing, water-film formation and localized wetting failure. These findings define the applicability and durability limits of superhydrophobic coatings for passive anti-icing protection of long-span transmission tower steel members.

## 1. Introduction

**Figure 1:**
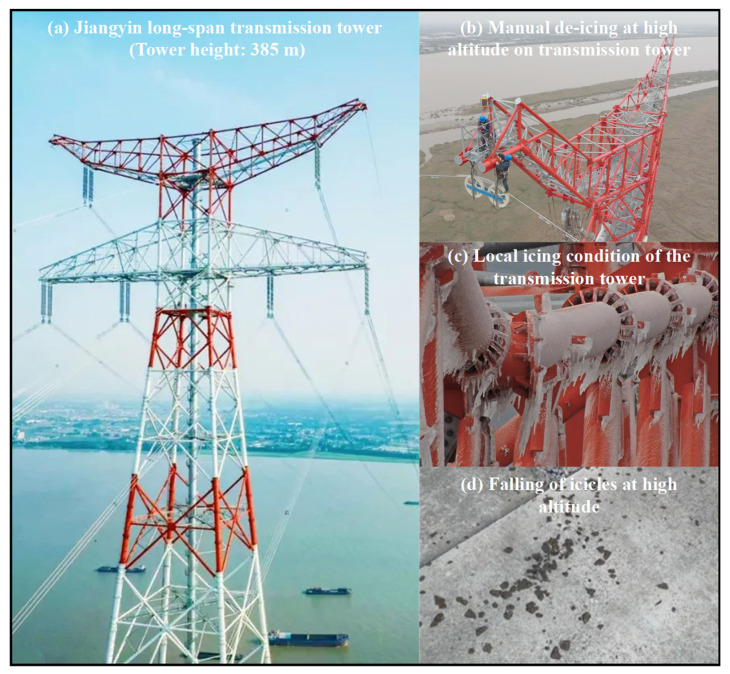
Icing conditions of a representative long-span transmission tower (Jiangyin Long-Span Transmission Tower, Jiangsu Province).

Long-span transmission towers are critical infrastructures for cross-river and cross-valley power transmission corridors, with their steel members continuously exposed to low temperatures, high humidity, strong winds, and supercooled droplets. For representative winter icing conditions in the middle-lower Yangtze River basin, transmission tower members are commonly exposed to subfreezing air temperatures of approximately −10 to 0 °C, high relative humidity generally exceeding 85%, and wind speeds that may reach 20 m/s during strong-wind icing events. Field statistics from southern China and the middle-lower Yangtze River basin show that transmission line icing is jointly driven by temperature, humidity, wind speed, liquid water content, and terrain [1], and is typically accompanied by persistent cold, near-saturated air, freezing rain or supercooled fog. These conditions cause severe and spatially nonuniform icing on high-altitude tower members over river surfaces [2]. The Jiangyin long-span transmission tower in Jiangsu Province, a representative project in this region, is persistently affected by the Jianghuai quasi-stationary front in winter. Supercooled droplet and fog concentrations at altitudes of 100–300 m are far higher than inland levels, resulting in sustained high icing risk. Icing increases structural dead load and windward area, amplifies wind-induced vibration and eccentric loading, and reduces the safety margin of members, joints and the coupled tower-line system [3], threatening the long-term reliable operation of transmission infrastructure and surrounding public safety (Figure 1). Accordingly, developing targeted anti-icing technologies for typical tower steel members has clear engineering significance.

Current transmission line anti-icing technologies are categorized into active deicing and passive anti-icing. Active methods (mechanical, electrothermal, chemical) work effectively for accessible line sections, but their application to high-altitude, geometrically complex tower members is limited by high energy consumption, operational difficulty and safety risks [4]. Passive anti-icing materials, by contrast, suppress ice growth by tuning surface wettability, interfacial thermal resistance and ice adhesion strength; they require no continuous energy input and thus show greater suitability for large-area, complex-shaped steel tower members. Theoretically, tower member icing is a multi-physics process coupling droplet transport, impingement, capture, phase change heat transfer and ice accumulation. Existing icing models for overhead conductors integrate liquid water content, wind speed, characteristic size, collision efficiency and freezing fraction into a unified framework, but they fail to fully account for the actual geometry and surface boundary conditions of tower components [5]. Steel tubes, angle steels and aged substrates in long-span towers cannot be simplified as smooth conductors or ideal cylinders, and their icing characteristics require systematic member-level experimental validation.

Most existing icing research focuses on overhead conductors. Icing prediction methods have evolved from empirical meteorological criteria to coupled physical-data-driven frameworks, with continuous improvements in model interpretability, temporal accuracy and adaptability to complex weather conditions [6,7,8,9,10]. However, these conclusions are mostly oriented to conductor scenarios and cannot be directly extended to angle steels and aged steel members with irregular cross-sections and surface states. Superhydrophobic surfaces achieve anti-icing effects by shortening droplet residence time, reducing the solid-ice real contact area and lowering ice adhesion strength. However, superhydrophobicity does not guarantee ideal icephobic performance: surface chemistry, roughness, condensation, frost formation and liquid penetration into microstructures all affect its actual anti-icing effectiveness [11,12,13]. For dynamic supercooled droplet impact, droplet rebound behavior is determined by the balance of impact dynamic pressure, spreading-retraction kinetics and freezing rate [14]. Existing studies also confirm that superhydrophobic anti-icing performance is highly dependent on test conditions, and must be evaluated under boundary conditions matching actual service environments [15].

Despite the above progress, three critical research gaps remain for the application of superhydrophobic anti-icing technology to long-span tower steel members. First, most existing tests rely on flat plates or small ideal specimens, which cannot reproduce the enhanced droplet capture effect at angle steel edges or the influence of corrosion defects on aged steel pipes [16]. Second, the coupling effect between member geometric features and coating microstructures is rarely considered, despite its non-negligible influence on interface wetting stability and anti-icing performance [17]. Third, systematic experimental data on the ice reduction performance and long-term stability of coatings under the combined conditions of strong wind, high humidity and continuous water film are still lacking at the member level [18].

To fill these research gaps, this study develops a multifactor coupled icing simulation system integrating low-temperature environment, wind field and supercooled droplets. Three typical tower members—aged plain circular steel tube, new galvanized circular steel tube, and new galvanized angle steel—are selected for comparative icing tests on bare and fluorosilicone resin-based superhydrophobic-coated surfaces. This work focuses on quantifying the combined effects of ambient temperature, wind speed, liquid water content, member cross-section geometry and substrate condition on coating ice reduction performance, providing experimental support for passive anti-icing design of long-span transmission towers in the Yangtze River basin. The main contributions of this work are summarized as follows: (1) comparative icing tests on three representative tower members reproduce the actual geometric and substrate characteristics of engineering components under service conditions typical of the Yangtze River basin; (2) the quantitative influences of temperature, wind speed, liquid water content and icing duration on coating anti-icing performance are systematically clarified; (3) the coupling mechanism of member geometry, substrate aging and coating wettability on anti-icing performance is revealed; and (4) the effective application scope of superhydrophobic coatings under high-humidity icing environments is defined to support their engineering application.

## 2. Icing Formation and Anti-Icing Mechanisms on Steel Surfaces

### 2.1. Ice Formation on Steel Member Surfaces

Ice formation on steel member surfaces is a complex physical process involving heat and mass transfer and phase change under coupled gas–liquid–solid interactions. When an airflow carrying supercooled water droplets impinges on the surface of a steel member at a temperature below the freezing point, the droplets generally undergo successive stages of collision, retention, freezing, and continued ice growth. Studies of design ice loads for overhead transmission lines have shown that the wind field, liquid water supply, and characteristic dimensions of line components must be considered simultaneously to explain differences in ice loads under varying environmental conditions [19]. For circular steel tubes and angle steels used in long-span transmission towers, geometric differences alter droplet impingement trajectories and the spatial distribution of ice. In addition, the high-humidity environment of the Yangtze River Basin regulates the icing growth rate by affecting the liquid water supply and thermal balance.

According to mass conservation, the ice-mass growth rate on a member surface can be expressed as the product of three principal efficiency factors and the incoming liquid water flux:

(1) $$\frac{dm}{dt}\;=\alpha_{1\;}\alpha_{2}\;\alpha_{3\;}w_{0}\;v_{0}\;A$$

where *w*_0_ is the liquid water content of the air, *v*_0_ is the incoming wind speed, *A* is the projected area of the member normal to the incoming flow, and α_1_, α_2_, and α_3_ are the collision, sticking, and freezing efficiencies, respectively.

#### 2.1.1. Collision Efficiency

Collision efficiency is defined as the ratio of the mass of droplets that impinge on the member surface to the total droplet mass passing through the projected area of the member. It is mainly governed by droplet inertia and member geometry. Droplets with greater inertia are less able to follow the airflow around the member and therefore exhibit a higher collision efficiency, whereas droplets with lower inertia are more readily deflected along the streamlines. Studies of atmospheric icing on non-rotating cylinders have shown that flow around the cylinder, droplet inertia, and the member characteristic dimension jointly govern droplet collection and ice distribution on the windward side [20].

Collision efficiency can therefore be characterized quantitatively by the Stokes number (St), defined as the ratio of the droplet relaxation time to the characteristic flow time:

(2) $$S_{t}=\frac{\rho_{w}\;d^{2}v_{0}}{18\mu D}\;\;$$

where *ρ*_w_ is the density of water, *d* is the mean droplet diameter, *μ* is the dynamic viscosity of air, and *D* is the characteristic dimension of the member.

For cylindrical members, the empirical Langmuir-Blodgett equation is commonly used to estimate collision efficiency, although its applicability depends on boundary conditions such as droplet size, wind speed, and member dimensions. Studies of empirical density equations for dry-growth ice on cylinders have shown that ice morphology and density are highly sensitive to environmental boundaries; therefore, consistency in the characteristic dimension must be maintained when empirical correction equations are applied [21].

This equation accounts for the influence of fluid viscosity on droplet collision through the Reynolds number (*Re*):

(3) $$\alpha_{1\;}=1-\exp(-S_{t}\cdot Re^{0.5})$$

The Reynolds number is based on the member characteristic dimension and is calculated as follows:

(4) $$Re=\frac{\rho_{a}\;v_{0}\;D}{\mu }\;$$

where *ρ*_a_ is the density of air.

For angle steels with asymmetric geometries, the edges and reentrant regions distort the local flow field and intensify droplet collection near the windward edges. Two-dimensional icing models for conductors and cables have shown that cross-sectional flow and local thermal boundary conditions strongly affect ice morphology. Therefore, an angle steel should not be treated simply as a circular tube with the same characteristic dimension [4].

A geometrically corrected expression for collision efficiency was adopted in this study:

(5) $$\alpha_{1,\mathrm{angle}\;}=C\cdot \alpha_{1,\mathrm{cylinder}}$$

where *α*_1,cylinder_ is the collision efficiency of a cylinder with the same characteristic dimension, and *C* is the shape correction coefficient.

#### 2.1.2. Capture Efficiency

Capture efficiency is defined as the ratio of the droplet mass retained by the surface to the droplet mass impinging on it. It is mainly governed by droplet-impact dynamics and surface wettability. When droplets impinge on surfaces with different wettabilities, their incident kinetic energy is redistributed among spreading, retraction, and viscous dissipation. Studies of droplet impact on surfaces ranging from hydrophilic to superhydrophobic have shown that wettability alters the spreading radius, retraction speed, and rebound probability [22]. When the residual kinetic energy exceeds the critical rebound energy, a droplet rebounds or splashes away from the surface; otherwise, it is retained.

Based on energy conservation, the capture efficiency can be expressed as a function of the Weber number (*We*):

(6) $$\alpha_{2}\;=1-\exp(-\frac{k}{We}\;)$$

where *k* is an empirical constant associated with surface wettability.

The Weber number based on droplet-impact speed is calculated as follows:

(7) $$We=\frac{\rho_{w}\;v_{i}^{2}\;d}{\sigma }\;$$

where *v_i_* is the normal speed of the droplet impinging on the surface, and *σ* is the surface tension of water.

#### 2.1.3. Freezing Efficiency

Freezing efficiency is defined as the ratio of the mass of droplets retained and frozen on the surface to the total mass of retained droplets. It is governed by the surface thermal balance. When droplets impinge on the member surface, the latent heat released during solidification is dissipated to the surrounding environment through thermal conduction and convective heat transfer. If the rate of heat dissipation exceeds the rate of latent heat release, the droplets freeze completely; otherwise, some of the water may drain from the surface before freezing. Studies of wind-turbine blade icing have shown that changes in ambient temperature and droplet size strongly affect ice mass and transitions between ice types. This behavior is also relevant to the effects of temperature and spray rate on ice mass investigated in this study [23].

According to the thermal balance equation, the latent heat released by droplets freezing per unit time is equal to the total heat dissipated through the surface:

(8) $$m_{f}\;L_{f}\;=hA(T_{s}\;-T_{a}\;)+\frac{kA(T_{s}\;-T_{w}\;)}{\delta }$$

where *m*_f_ is the mass of droplets frozen per unit time, *L*_f_ is the latent heat of fusion of water, *h* is the convective heat-transfer coefficient, *T*_s_ is the ice-surface temperature, *T*_a_ is the ambient temperature, *k* is the thermal conductivity of ice, *T*_w_ is the droplet temperature, and *δ* is the ice-layer thickness.

The freezing efficiency can be expressed as the ratio of the actual frozen mass to the theoretical maximum frozen mass:

(9) $$\alpha_{3}\;=\frac{m_{f}}{m_{i\;}}\;\;$$

where *m*_i_ is the mass of droplets impinging on the surface per unit time.

Under the high-humidity conditions of the Yangtze River Basin, the liquid water supply is relatively high, resulting in a large mass of droplets impinging on the surface per unit time and correspondingly greater latent heat release. This can increase the ice-surface temperature and alter the freezing efficiency. Previous icing studies have likewise shown that ambient temperature and droplet size strongly affect ice growth and transitions between ice types, which is consistent with the effects of temperature and spray rate on ice mass observed in this study [6].

#### 2.1.4. Relationship Between Ice Type and Environmental Parameters

Ice type is determined primarily by ambient temperature and liquid water supply, reflecting differences in the thermal balance. Makkonen’s icing model integrates glaze ice, rime ice, icicles, and wet snow within a unified mass- and energy-conservation framework, indicating that ice-type classification should facilitate interpretation of the thermal balance and water-collection mechanisms [18]. A dimensionless ice-type criterion (λ) can be introduced to classify icing quantitatively:

(10) $$\lambda =\frac{w_{0}\;v_{0}\;L_{f}}{h(0-T_{a}\;)}\;\;$$

Based on the value of λ, icing can be divided into three basic types. Glaze ice forms when λ > 1.0, mixed ice forms when 0.3 < λ ≤ 1.0, and rime ice forms when λ ≤ 0.3. These ice types differ in density and mechanical properties. By varying temperature, wind speed, and spray rate, the experiments in this study covered the principal ranges associated with representative icing environments, allowing the icing behavior of different steel members and the ice-reduction performance of the superhydrophobic coating to be compared.

### 2.2. Anti-Icing Mechanisms of Superhydrophobic Coatings

The anti-icing effect of a superhydrophobic surface arises from the combined action of its micro/nanoscale roughness and low-surface-energy modification. The associated mechanisms correspond to the ice-growth processes described in Section 2.1: modifying droplet-impact dynamics reduces the capture efficiency; the interfacial trapped-air layer increases thermal resistance and delays freezing; and reducing the actual ice-solid contact area weakens ice adhesion. The Cassie equation indicates that the composite contact state should not be interpreted solely as a geometric relationship but should instead be considered together with the actual solid–liquid contact fraction, the stability of trapped air, and the surface condition [24]. These three coupled mechanisms jointly inhibit ice formation, delay ice growth, and facilitate ice shedding.

#### 2.2.1. Theoretical Basis of Wettability on Superhydrophobic Surfaces

Two representative wetting states can occur on rough surfaces: the Cassie–Baxter composite contact state and the Wenzel fully wetted state. The porous-surface wetting model proposed by Cassie and Baxter indicates that a droplet can form composite contact while being supported jointly by solid asperities and trapped air, thereby producing a large apparent contact angle [21]. On a superhydrophobic surface, the droplet remains in composite contact above the micro/nanostructures, while a substantial amount of air is trapped within the structural gaps, forming an interfacial system in which solid, air, and liquid phases coexist. The apparent contact angle is described by the Cassie–Baxter equation:

(11) $$\cos\theta^{*}=f_{s}\;\cos\theta_{s}\;+f_{a}\;\cos\theta_{a}$$

where θ* is the apparent contact angle, *θ*_s_ is the intrinsic contact angle of the corresponding smooth solid surface, *θ*_a_ is the contact angle associated with the air–water interface, *f*_s_ is the fraction of the projected area occupied by solid–liquid contact, and *f*_a_ is the fraction occupied by air–liquid contact, with *f*_s_ + *f*_a_ = 1.

Because the contact angle associated with the air fraction is approximately 180°, substitution into Equation (11) gives the simplified form of the Cassie–Baxter model:

(12) $$\cos\theta^{*}=f_{s}\;(\cos\theta_{s}\;+1)-1$$

Equation (12) shows that when a low-surface-energy material produces an intrinsic contact angle greater than 90°, a smaller solid–liquid contact fraction results in a larger apparent contact angle and weaker interfacial droplet adhesion. When a droplet fully penetrates the rough surface structure, its wetting behavior is described by the Wenzel model. Wenzel’s investigation of wetting resistance on rough surfaces showed that roughness amplifies the intrinsic wettability of a material and produces complete contact between the droplet and the solid surface [22]. The apparent contact angle is expressed as follows:

(13) $$\cos\theta^{*}=r\cos\theta_{s}\;$$

where *r* is the surface roughness factor, defined as the ratio of the actual solid surface area to its projected area.

Stable anti-icing performance of a superhydrophobic surface requires the Cassie–Baxter state to remain intact. Once a transition from the Cassie state to the Wenzel state occurs, droplet adhesion increases substantially and the anti-icing performance of the coating deteriorates. Studies of flexible anti-icing coatings with abrasion and corrosion resistance have shown that mechanical stability, interfacial integrity, and corrosion protection under long-term service conditions also affect anti-icing performance. A high static contact angle alone therefore cannot ensure stable anti-icing behavior [25].

#### 2.2.2. Droplet-Rebound Mechanism and Regulation of Capture Efficiency

The impingement of supercooled water droplets on a member surface is the initial stage of ice formation. A superhydrophobic coating directly reduces the surface capture efficiency by enhancing droplet rebound, which constitutes its primary dynamic ice-reduction mechanism. Experiments involving supercooled droplets impinging on textured superhydrophobic surfaces have shown that rebound is governed by the competition among impact dynamic pressure, surface-structure dimensions, and freezing time [24]. The droplet-impact process follows energy conservation: part of the incident kinetic energy is converted into surface energy, part is dissipated by viscous effects, and the remaining energy drives droplet retraction and rebound.

The normal restitution coefficient of a droplet (e) is defined as the ratio of the rebound speed to the incident speed and can be expressed as follows:

(14) $$e=\sqrt{\frac{E_{k}\;-E_{d}\;-\Delta E_{s}}{E_{k}\;}}\;\;\;$$

where *E*_k_ is the incident kinetic energy of the droplet, *E*_d_ is the energy dissipated by viscous effects during impact, and Δ*E*_s_ is the change in surface energy during droplet spreading and retraction.

On conventional hydrophilic or hydrophobic surfaces, droplets exhibit strong adhesion after spreading, insufficient retraction force, and a high proportion of viscous energy dissipation. Consequently, the restitution coefficient approaches zero, and nearly all droplets are retained by the surface. On a Cassie-state superhydrophobic surface, the solid–liquid contact area and interfacial work of adhesion are small. Droplets can therefore retract rapidly after spreading, with a reduced proportion of viscous dissipation and an increased restitution coefficient. Studies of the effects of surface topology on icephobicity have shown that microstructure dimensions and connectivity determine whether a superhydrophobic coating provides an effective icephobic advantage [16]. According to the definition of capture efficiency in Section 2.1.2, capture efficiency is quantitatively related to the restitution coefficient: the fraction of droplet mass that does not rebound after impact is retained by the surface. Capture efficiency can therefore be expressed as a decreasing function of the restitution coefficient. This mechanism acts directly on the *α*_2_ term in the icing-growth model and reduces the mass of droplets participating in the freezing process at the source.

#### 2.2.3. Interfacial Thermal-Insulation Mechanism and Delayed Freezing

For droplets that fail to rebound and remain on the surface, the superhydrophobic coating can delay freezing through the thermal-insulation effect of the interfacial air layer, thereby reducing the freezing efficiency. In the Cassie–Baxter state, heat transfer between the droplet and the substrate occurs through two parallel pathways: the solid–liquid contact points and the air-liquid interfaces. Anti-icing heat-transfer models for biomimetic microstructured superhydrophobic surfaces have shown that the microstructure geometry, trapped-air layer, and actual solid–liquid contact area jointly modify the heat-transfer pathways during droplet freezing [26]. The equivalent interfacial thermal resistance (*R*_eq_) can be expressed as follows:

(15) $$R_{\mathrm{eq}}\;=\frac{\;\;1}{\frac{f_{s}\;k_{s}}{\delta_{s}\;}\;\;+\frac{\;f_{a}\;k_{a}}{\delta_{a}}}\;$$

where *k*_s_ is the thermal conductivity of the solid material, *k*_a_ is the thermal conductivity of air, *δ*_s_ is the characteristic height of the surface microstructures, and *δ*_a_ is the effective thickness of the interfacial trapped-air layer.

Because the thermal conductivity of air is much lower than that of metallic materials, the equivalent interfacial thermal resistance can be higher than that of a smooth metal surface even when the air-liquid contact fraction is not particularly large. The increased thermal resistance directly reduces the heat-transfer rate between the droplet and the cold substrate, thereby extending the time required for the droplet to transition from its initial supercooled state to complete freezing. Based on the lumped-capacitance approach, the freezing time of an individual droplet is positively correlated with the interfacial thermal resistance and can be derived from the fundamental phase-change heat-transfer equation as follows:

(16) $$\tau_{f}=C_{g}\frac{\rho_{w}L_{f}dR_{\mathrm{eq}}}{T_{f}-T_{s}}$$

where *τ*_f_ is the time required for complete droplet freezing, *C*_g_ is a dimensionless geometric coefficient associated with the droplet shape and interfacial heat-transfer area, *ρ*_w_ is the density of water; *L*_f_ is the latent heat of fusion of water, *d* is the diameter of the droplet, *T*_f_ is the freezing temperature of water; and *T*_s_ is the substrate surface temperature.

The longer freezing time provides a time window during which droplets can detach from the surface under gravity or wind-induced shear. Some droplets may roll off before freezing completely, further reducing the actual ice mass. This mechanism corresponds to the *α*_3_ term in the icing-growth model and suppresses ice growth from the perspective of phase-change kinetics.

#### 2.2.4. Reduced-Contact Mechanism and Weakened Ice Adhesion

After droplets have frozen on a superhydrophobic surface, the composite interfacial structure associated with the Cassie state can continue to reduce icing, primarily by decreasing the ice-solid interfacial adhesion strength. Adhesion between ice and a solid surface originates from intermolecular interactions at the interface, and its macroscopic strength is proportional to the true contact area.

An ice layer formed in the Cassie–Baxter state is molecularly bonded to the substrate only at solid–liquid contact points, whereas most of the interface consists of ice-air contact. The apparent ice adhesion strength can be expressed as follows:

(17) $$\tau_{\mathrm{ice}}\;=f_{s}\;\cdot \tau_{\mathrm{ice},0}\;$$

where *τ*_ice_ is the apparent adhesion strength of ice on the rough surface, and *τ*_ice,0_ is the intrinsic adhesion strength of ice on the corresponding smooth solid surface.

The reduction in adhesion strength allows the ice layer to detach more readily under wind loading, structural vibration, gravity, or thermal stress. This reduces long-term ice mass and the probability of severe icing hazards. The mechanism supplements the passive anti-icing effect of superhydrophobic coatings and is particularly relevant under intermittent icing conditions.

#### 2.2.5. Instability of the Cassie State and the Failure Boundary of Superhydrophobic Anti-Icing

The anti-icing performance of a superhydrophobic coating is not unconditional; its fundamental prerequisite is the stability of the Cassie–Baxter contact state. When an external driving force exceeds a critical value, droplets penetrate the trapped-air barrier and completely infiltrate the micro/nanostructures, resulting in a Cassie-to-Wenzel transition and a rapid deterioration in anti-icing performance. Studies of durable anti-icing slippery surfaces have shown that pore size, porosity, and liquid- or air-storage structures strongly influence interfacial stability. The pressure-bearing capacity of the microstructures is therefore an important factor in determining the anti-icing failure boundary [27].

Based on interfacial thermodynamic equilibrium, the critical pressure for the state transition can be derived from the Laplace equation:

(18) $$P_{c}\;=-\frac{\;2\sigma \cos\theta_{s}}{d_{m}}\;\;$$

where *P*_c_ is the critical pressure for destabilization of the Cassie state, *σ* is the surface tension of water, and *d*_m_ is the characteristic spacing of the surface microstructures.

Under actual icing conditions, the driving forces that induce this transition arise primarily from two sources. The first is the dynamic pressure generated by high-speed droplet impact. Higher wind speeds and larger droplets produce greater impact dynamic pressures, making the critical pressure more likely to be exceeded. The second is the hydrostatic pressure associated with continuous water-film formation under a high liquid water supply. Continuous droplet accumulation increases the interfacial pressure and drives water into the surface structures. In addition, extremely low temperatures increase the viscosity of water and weaken droplet retraction, thereby accelerating destabilization of the Cassie state.

These failure mechanisms indicate that the ice-reduction performance of superhydrophobic coatings is bounded by specific environmental parameters. For the high-humidity service environment of the Yangtze River Basin, where multiple types of icing may occur, systematic experiments are required to quantify the effective operating range of the coating. Defining this range is one of the primary objectives of the present experimental study. In summary, superhydrophobic coatings achieve anti-icing performance through the coupled mechanisms of capture-efficiency regulation, delayed freezing, and weakened ice adhesion. These mechanisms correspond to the key parameters in the icing-growth model, and their performance is jointly constrained by environmental conditions and surface state. Recent studies of superhydrophobic coatings combining corrosion protection, passive anti-icing, and active deicing have also shown that engineered anti-icing surfaces must simultaneously account for wettability, thermal response, mechanical stability, and substrate protection rather than pursuing a single contact-angle criterion [28]. This theoretical framework provides a unified basis for the subsequent experimental design, interpretation of results, and analysis of the underlying mechanisms.

## 3. Icing Test Plans

Ice formation on steel members of long-span transmission towers is a complex multiphysics process involving low-temperature phase change, wind-driven droplet transport, and continuous liquid water supply. Field measurements are constrained by the stochastic nature of meteorological conditions and the difficulties associated with high-altitude operations, making systematic parametric and quantitative investigations difficult. To examine the ice-reduction mechanisms of superhydrophobic surfaces and the governing effects of environmental parameters, controlled icing tests were conducted using an independently developed multifactor coupled icing simulation system. Representative icing conditions in the Yangtze River Basin were reproduced by precisely regulating key environmental parameters, including temperature, wind speed, and liquid water content. The icing evolution of uncoated and superhydrophobic-coated surfaces on different steel members was then compared to provide experimental support for theoretical model validation and engineering design.

### 3.1. Development of a Multifactor Coupled Icing Simulation System

**Figure 2:**
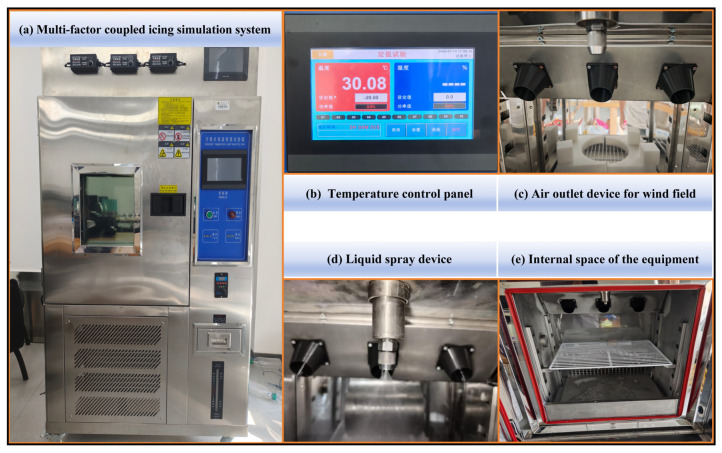
Independently developed multifactor coupled icing simulation system.

Ice formation on steel members of long-span transmission towers results from the coupled effects of low-temperature phase change, wind-driven transport of supercooled water droplets, and continuous liquid water supply. Because field measurements are restricted by meteorological variability and high-altitude operating conditions, systematic parametric investigations are difficult to perform. To characterize icing evolution and the ice-reduction mechanisms of superhydrophobic surfaces, a multifactor coupled icing simulation system was independently developed. A commercial environmental icing chamber was used as the main platform and integrated with refrigeration and temperature control, circulating airflow, high-pressure atomization, and data acquisition modules. The resulting low-temperature, wind-field, and supercooled-droplet simulation system enabled independent regulation and coordinated coupling of temperature, wind speed, and liquid water content. It could stably reproduce representative winter icing environments in the Yangtze River Basin, including freezing rain, supercooled fog, and mixed icing, thereby providing controlled environmental conditions for comparative icing tests on different steel members, as shown in Figure 2.

The main unit was a LISK-HW150C environmental icing chamber with an effective internal workspace of 0.5 m × 0.4 m × 0.3 m, which was supplied by LISK Instrument Equipment (Nanjing) Co., Ltd. (Nanjing, China). The chamber could accommodate representative specimens, including angle-steel segments and short steel-tube segments, and therefore met the dimensional requirements for small-scale investigations of member-level icing mechanisms. Its temperature control range was −40 to 60 °C, with a display resolution of 0.1 °C and a temperature deviation within the working region of no more than ±0.5 °C. This range covered the representative formation temperatures from rime ice to glaze ice. The chamber was equipped with a multilayer sealed observation window, an internal cold-light source, and test-lead interfaces, enabling dynamic observation of the icing process and connection of multiple sensors. Experimental parameters could be stored in real time to support subsequent data traceability and repeatability assessment.

The low-temperature control unit employed a fully enclosed compressor refrigeration system and a recirculating air-duct configuration. An electric heating compensation unit and a proportional–integral–derivative closed-loop control algorithm were used in conjunction with PT100 platinum resistance temperature sensors manufactured by Shanghai Automation Instrument Co., Ltd. (Shanghai, China) installed within the test region to acquire temperature data in real time. This configuration maintained a uniform and stable temperature field inside the chamber and minimized interference from temperature fluctuations with the onset of freezing and the compactness of the ice layer.

The wind-field control unit consisted of a variable-frequency axial-flow fan and flow-straightening grids. The wind speed could be continuously adjusted from 0 to 20 m/s, covering the representative winter wind-speed range over the Yangtze River. After flow conditioning, the airflow exhibited relatively low turbulence intensity, allowing the transport and impact kinetic energy of supercooled droplets to be simulated under different wind speeds. The system also reproduced local flow distortion and droplet accumulation near the edges of non-streamlined members.

The spray control unit comprised an array of high-pressure air-atomizing nozzles arranged at equal intervals upstream of the windward side of the specimens. Deionized water and compressed air were mixed and atomized inside the nozzles. The droplets rapidly cooled to a supercooled state while being transported by the airflow through the low-temperature chamber. By adjusting the air-to-liquid pressure ratio, the median volume diameter of the droplets was maintained at approximately 50 μm. The spray rate could be varied continuously to provide spray rates of 0.5–2.5 L/min, corresponding to different liquid water supply intensities. Deionized water was used as the spraying medium to prevent impurity ions from affecting surface wettability and the physical properties of the ice layer.

A central touchscreen control unit enabled coordinated regulation of multiple parameters. Before each formal test, a precooling stabilization stage was applied to establish sufficient thermal equilibrium between the specimen and the chamber environment. After the prescribed steady-state conditions were reached, the wind-field and spray systems were activated simultaneously, and timing was initiated. This procedure prevented deviations in the initial boundary conditions caused by premature water accumulation, early freezing, or nonuniform spraying and ensured consistent test conditions and data repeatability among parallel specimens.

### 3.2. Test Conditions

To systematically investigate the coupled effects of member cross-sectional geometry, surface wettability, and environmental parameters on the icing characteristics of steel members, the experimental variables were defined in terms of specimen properties and environmental conditions. Comparative icing tests were conducted using the controlled-variable method. Three representative steel members from long-span transmission towers were selected, and each member was tested with both its original uncoated surface and a superhydrophobic-coated surface. Ambient temperature, wind speed, and liquid water content were selected as the principal environmental factors. Multiple levels were established to cover representative winter icing conditions in the Yangtze River Basin, and observations were conducted at several time points to characterize the complete evolution of ice growth.

**Figure 3:**
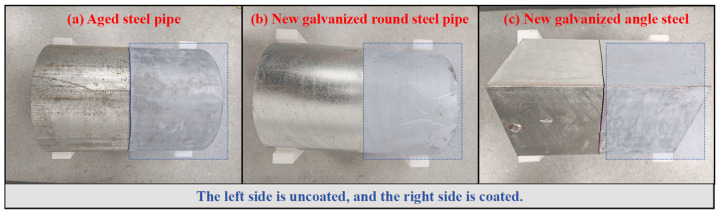
Representative test specimens and application of the superhydrophobic coating.

Based on engineering requirements, three representative member types were selected: an aged circular steel tube, a new hot-dip galvanized circular steel tube, and an equal-leg angle steel, as shown in Figure 3. The specimens were obtained from full-scale steel members used in practical long-span transmission tower engineering rather than from geometrically scaled laboratory models. Owing to the limited effective workspace of the icing chamber, the full-scale members were cut into 300 mm long segments for the icing tests while retaining their original cross-sectional dimensions. The circular steel tubes had dimensions of Φ200 × 5 mm, while the equal-leg angle steels had dimensions of L160 × 10 mm. These specimen dimensions were compatible with the effective chamber space, allowing for sufficient development of the flow field around each specimen and minimizing interference from wall boundary effects with droplet impingement. The tested members were selected from steel product forms commonly used in transmission tower engineering. Transmission tower members are generally fabricated from hot-rolled carbon structural steels or low-alloy high-strength structural steels, such as Q345 -series steels. These steels are Fe-based alloys in which C, Si, Mn, P, and S are the principal controlled chemical elements, and the higher-strength grades are typically characterized by increased Mn and Si contents and higher yield strength. In the present study, the aged circular steel tube represented a service-degraded tower-member surface, whereas the new galvanized circular steel tube and the new galvanized angle steel represented commonly used hot-dip galvanized tower members. Therefore, the three specimens covered both circular-tube and angle-steel structural forms as well as aged and newly galvanized surface states relevant to long-span transmission towers.

**Figure 4:**
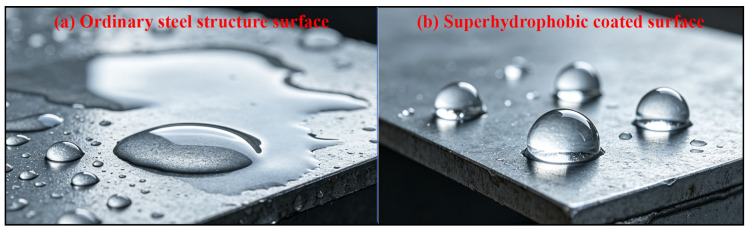
Water-droplet behavior on uncoated and superhydrophobic-coated steel surfaces.

**Figure 5:**
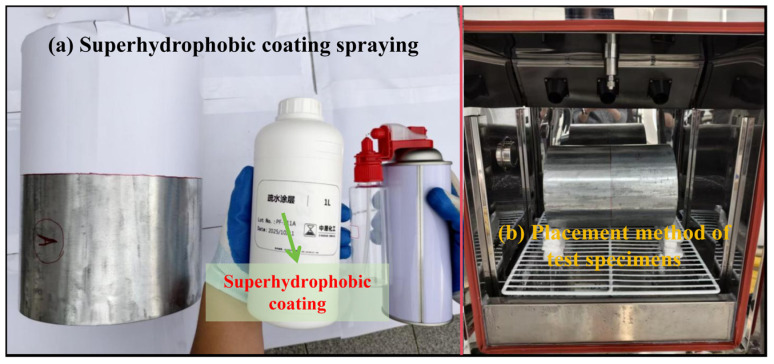
Application of the superhydrophobic coating and preparation of the specimen arrangement.

As shown in Figure 4, each member type included an uncoated reference group and a superhydrophobic-coated comparison group. The superhydrophobic coating used in this study was XN-204A abrasion-resistant superhydrophobic spray solution, a commercial fluorosilane-polymer-based coating sourced from Shenzhen Weijing New Material Technology Co., Ltd., Shenzhen, China. According to the technical data sheet, the coating has a solid content of 12%, a density of 0.8 g/mL, and butyl acetate as the solvent. The product exhibits a water contact angle greater than 150° and a rolling angle lower than 5°, with a coating adhesion grade of 0, and is applicable to metallic substrates. As shown in Figure 5, the coating was applied and cured using a standardized high-pressure spraying process. Before spraying, the coating solution was thoroughly stirred until no visible sediment remained and was used without dilution or mixing with other solvents. Before coating or testing, all specimens were ultrasonically cleaned in anhydrous ethanol to remove surface dust, grease and loose contaminants, and were then dried before subsequent treatment. For the aged circular steel tube, only loose surface contaminants were removed, while the aged surface condition was retained to represent long-term service exposure. The uncoated reference specimens underwent the same cleaning and drying procedure as the coated specimens, but no superhydrophobic coating was applied. Before testing, surface wettability was characterized through contact-angle measurements to ensure consistent surface properties among the specimens. Three parallel specimens were prepared for each group to reduce random variability in the experimental data. Considering the environmental characteristics of winter icing on long-span transmission towers in the Yangtze River Basin, ambient temperature, incoming wind speed, and liquid water content were selected as the environmental control variables for the three representative member types, and multiple factor levels were established. Several consecutive observation times were specified for each condition to determine the time-dependent evolution of ice mass. Single-factor effects were investigated by varying one parameter at a time while maintaining the remaining parameters constant. Each test condition was repeated three times under identical environmental settings. Unless otherwise stated, quantitative results are reported as the arithmetic mean ± standard deviation (SD) of the three repeated measurements. The SD was used to characterize the dispersion of repeated measurements and is shown as error bars in the corresponding result figures. The detailed experimental variables and their levels are listed in Table 1.

### 3.3. Experimental Procedure

All icing tests followed a standardized procedure, with the boundary conditions at each stage strictly controlled to ensure the reliability and repeatability of the measured data. Before coating or testing, all specimens were ultrasonically cleaned in anhydrous ethanol to remove surface dust, grease and loose contaminants, and were then dried. The uncoated reference specimens followed the same cleaning and drying procedure as the coated specimens. After surface preparation, each specimen was weighed using an electronic balance to determine and record its initial mass. The low-temperature control system was then activated, and the test chamber was adjusted to the target temperature and maintained under stable precooling conditions to ensure a uniform internal temperature field. Each specimen was mounted vertically on a purpose-built fixture, with its longitudinal axis perpendicular to the incoming airflow. The specimen was held under these conditions until thermal equilibrium was established between its surface and the surrounding environment, thereby minimizing the influence of the initial temperature difference on the icing process.

**Figure 6:**
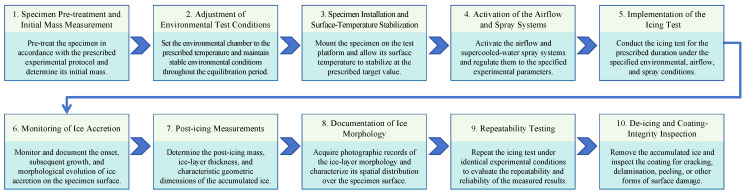
Flowchart of the complete icing test procedure.

After steady-state conditions were reached, the wind-field and high-pressure atomizing spray systems were activated simultaneously, and timing was initiated to begin the formal icing stage. Throughout the test, an image-acquisition system operating through the observation window continuously recorded ice nucleation, growth, and morphological evolution. At each prescribed observation time, the airflow and spray systems were rapidly suspended, and the specimen was removed to determine its total mass after icing. Ice-layer thickness and characteristic geometric dimensions were measured at multiple locations using a vernier caliper, and the macroscopic ice morphology was photographed. All measurements were completed rapidly under low-temperature conditions to minimize errors caused by ice melting. Three parallel tests were conducted for each condition. After all tests had been completed, the ice layer was removed from the specimen surface, and the coated specimens were visually checked to identify obvious coating detachment or macroscopic surface damage. This post-icing check was used only as a qualitative observation and was not intended to quantify wear resistance, adhesion strength, abrasion resistance, or microscopic surface changes. The overall experimental procedure is shown in Figure 6.

### 3.4. Evaluation Metrics and Methods

To quantitatively characterize icing evolution on steel member surfaces and the ice-reduction performance of the superhydrophobic coating, an evaluation framework was established in terms of total ice mass, growth kinetics, ice-reduction performance, and spatial distribution. Each metric was calculated from the measured experimental data using the equations defined below to ensure objective and comparable evaluations.

#### 3.4.1. Ice Mass

Ice mass represents the total amount of ice accumulated on the specimen surface. It was calculated as the difference between the total specimen mass after icing and its initial mass and provides the most direct measure of the extent of icing:

(19) $$m_{\mathrm{ice}}\;=m_{t}\;-m_{0}$$

where *m*_ice_ is the mass of ice accumulated on the specimen surface, *m*_t_ is the total specimen mass after icing, and *m*_0_ is the initial net mass of the specimen.

#### 3.4.2. Average Ice-Growth Rate

The ice-growth rate characterizes the growth kinetics of the ice layer at different stages and corresponds to the mass-growth rate in the icing model presented in Section 2. The average growth rate over a given time interval was calculated from the increase in ice mass per unit time:

(20) $$v_{\mathrm{ice}}\;=\frac{\Delta m_{\mathrm{ice}}}{\Delta t}\;\;$$

where *v*_ice_ is the average ice-growth rate over the corresponding time interval, Δ*m*_ice_ is the increase in ice mass during Δ*t*, and Δ*t* is the measurement time interval.

#### 3.4.3. Ice Reduction Rate

The ice reduction rate is the principal dimensionless metric used to evaluate the effectiveness of the superhydrophobic coating. It was defined as the relative difference in ice mass between the uncoated reference specimen and the coated specimen under the same test condition:

(21) $$\eta =\;\;\frac{m_{\mathrm{ref}}\;-m_{\mathrm{coat}}}{m_{\mathrm{ref}}}\;\;\;\times 100\%$$

where *η* is the ice reduction rate of the superhydrophobic coating, *m*_ref_ is the ice mass of the uncoated reference specimen, and *m*_coat_ is the ice mass of the superhydrophobic-coated specimen under the same condition.

#### 3.4.4. Ice-Layer Thickness and Inhomogeneity Coefficient

For each specimen, ice-layer thickness was measured at three representative circumferential locations: the windward impingement side, the lateral side and the leeward side. These locations were selected to represent the principal icing zones formed under directional airflow, including direct droplet impingement, lateral runoff or edge growth, and leeward recirculation. For the angle-steel specimens, the lateral measurement was taken near the edge region where local ice accumulation was most pronounced. The three-point method was used to provide a consistent engineering index for comparing ice-thickness distribution among different specimens and test conditions, rather than to reconstruct the complete circumferential ice profile. Therefore, the inhomogeneity coefficient was interpreted as a relative comparative parameter under identical measurement rules.

The mean ice-layer thickness was calculated as the arithmetic mean of the thicknesses measured at all locations:

(22) $$\bar{\delta }=\frac{1}{n}\;\;\sum_{i=1}^{n}\delta_{i}\;\;$$

where $\bar{\delta }$ is the mean ice-layer thickness, *δ_i_* is the ice-layer thickness at the *i*-th measurement location, and *n* is the total number of measurement locations. In this study, n = 3, corresponding to the windward, lateral, and leeward sides.

The ice-thickness Inhomogeneity coefficient was defined as the ratio of the standard deviation of the measured ice-layer thicknesses to their mean value and was used to characterize the relative circumferential nonuniformity of the ice layer:

(23) $$C_{u\;}=\frac{S_{\delta }}{\bar{\delta }}\;$$

The standard deviation of ice-layer thickness was calculated as follows:

(24) $$S_{\delta }\;=\frac{1}{n-1}\sum_{\;i=1}^{n}\;{\left(\delta_{i}\;-\bar{\delta }\right)}^{2}$$

where *C*_u_ is the ice-thickness Inhomogeneity coefficient, and *S*_δ_ is the sample standard deviation of the ice-layer thicknesses measured at the selected locations. A larger Inhomogeneity coefficient indicates a more uneven circumferential distribution of the ice layer and a more pronounced eccentric loading effect on the member. It should be noted that this method may underestimate local extreme ice thickness on sharp-edged members, especially when icicles develop near the angle-steel edges. Therefore, the calculated inhomogeneity coefficient was evaluated together with the recorded macroscopic ice morphology to support the interpretation of nonuniform icing behavior.

## 4. Effects of the Superhydrophobic Coating on the Icing Characteristics of Different Members

This section examines three representative steel members used in long-span transmission towers. Systematic comparative tests were conducted using the coupled low-temperature, wind-field, and supercooled-droplet icing simulation system to characterize icing evolution on the original reference surfaces and fluorosilicone resin-based superhydrophobic-coated surfaces. The three member types were an aged plain circular steel tube, a new galvanized circular steel tube, and a new galvanized angle steel. The detailed parameter settings listed in Section 3.2 were adopted as the test conditions. A control variable approach was used to investigate the effects of ambient temperature, incoming wind speed, spray rate, and icing duration. The icing evolution under different conditions was further interpreted using an icing growth kinetics model mainly dominated by interfacial thermal resistance. All evaluation indicators were calculated using the equations defined in Section 3.4. Identical test conditions were applied to the three member types to ensure consistent boundary conditions for horizontal comparison. The icing characteristics and coating-induced ice-reduction performance of each member are analyzed sequentially, followed by an examination of how member cross-sectional geometry and substrate surface condition affect the ice-reduction performance of the superhydrophobic coating.

### 4.1. Aged Circular Steel Tube

**Figure 7:**
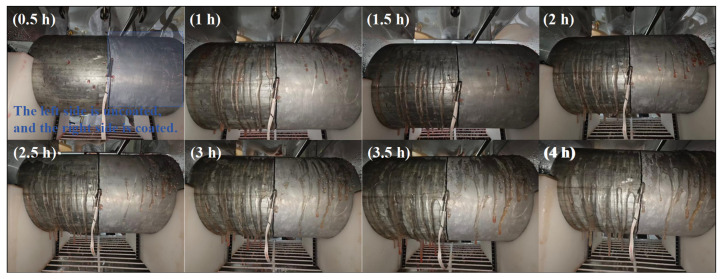
Comparison of anti-icing performance between uncoated and superhydrophobic-coated aged circular steel tubes.

The aged circular steel tube represented the condition of a primary tower member after long-term service. Its galvanized layer had deteriorated and exhibited localized corrosion, resulting in greater surface roughness and higher intrinsic surface energy than those of the new members. Consequently, it exhibited a higher droplet capture efficiency and a lower threshold for icing initiation. The icing characteristics of this member under the reference condition are shown in Figure 7.

**Figure 8:**
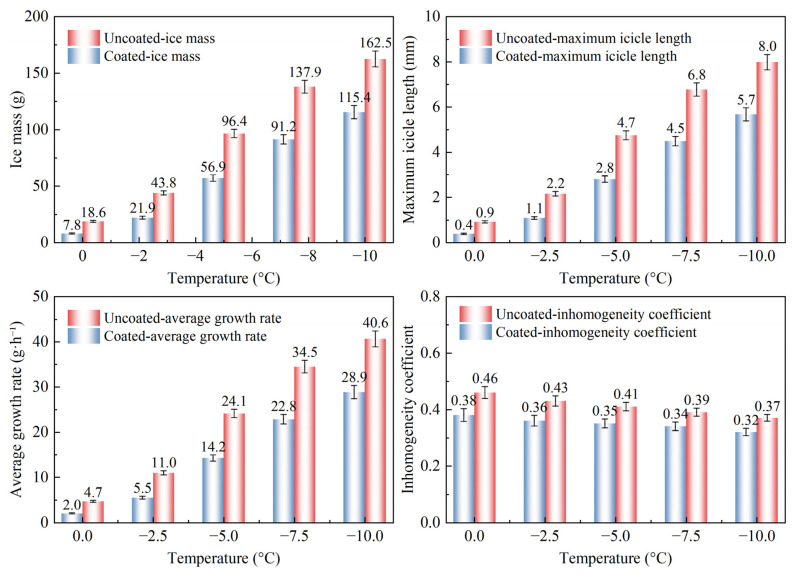
Comparison of icing characteristics of the aged circular steel tube at different ambient temperatures.

**Figure 9:**
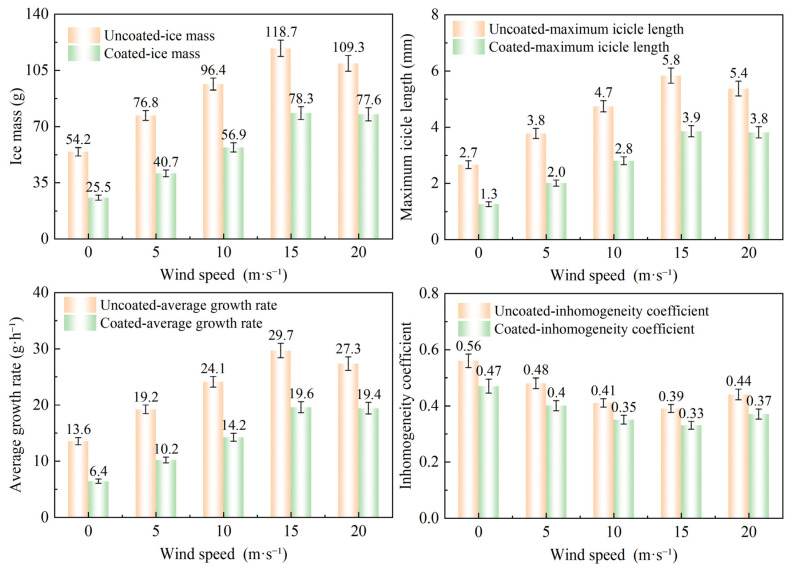
Comparison of icing characteristics of the aged circular steel tube at different wind speeds.

**Figure 10:**
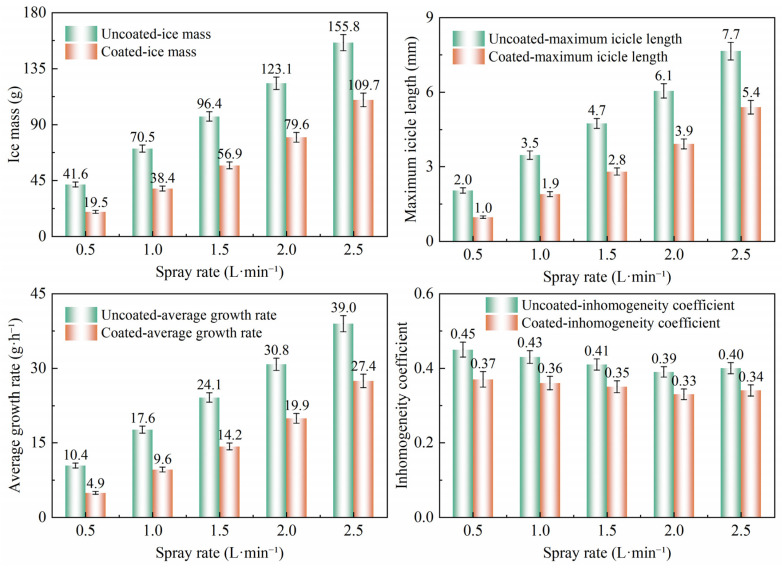
Comparison of icing characteristics of the aged circular steel tube at different spray rates.

**Figure 11:**
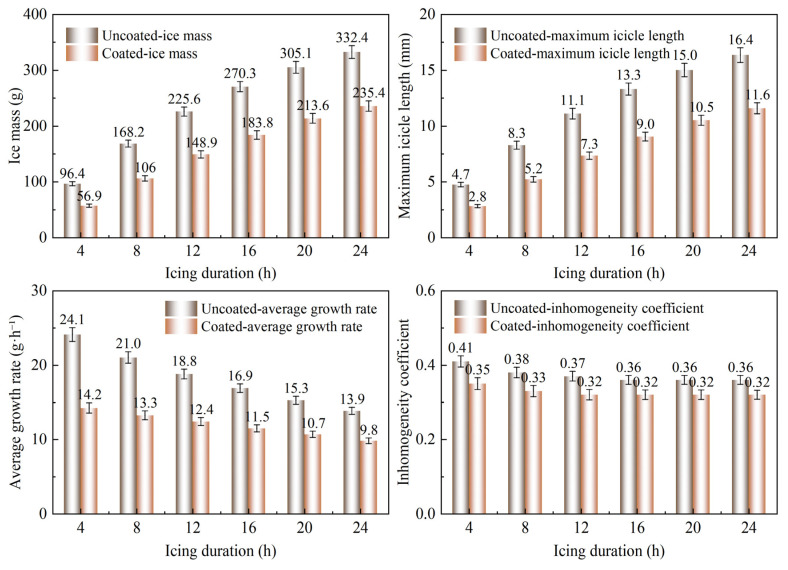
Comparison of icing characteristics of the aged circular steel tube at different icing durations.

The icing behavior of the uncoated reference surface agreed well with the theoretical model. As shown in Figure 8, ice mass increased monotonically with a higher degree of supercooling. When the ambient temperature decreased from 0 to −10 °C, the ice mass increased by 774%. This trend was primarily attributed to the greater heat-transfer temperature difference, which markedly increased the freezing efficiency (*α*_3_). Meanwhile, the inhomogeneity coefficient decreased from 0.46 to 0.37. At a higher degree of supercooling, droplets froze more rapidly and exhibited less surface flow, resulting in a more uniform circumferential ice distribution. As shown in Figure 9, ice mass initially increased and subsequently decreased with increasing wind speed. The maximum value occurred at 15 m/s and was 119% higher than that under the no-wind speed condition. This increase was primarily governed by enhanced droplet inertia and a corresponding increase in the collision efficiency (*α*_1_). At 20 m/s, the ice mass decreased slightly due to increased droplet splashing and secondary detachment of the ice layer. As shown in Figure 10, ice mass increased approximately linearly with spray rate. When the spray rate increased from 0.5 to 2.5 L/min, the ice mass increased by 275%. Within this range, the freezing efficiency had not reached saturation, and ice growth was mainly governed by the liquid water flux. As shown in Figure 11, ice mass exhibited a characteristic decelerating growth pattern over prolonged icing durations. From 4 to 24 h, the average ice growth rate decreased by 43%, consistent with the thermal resistance growth mechanism proposed in the classical Makkonen icing model. After the application of the superhydrophobic coating, the ice mass, growth rate, and ice-layer thickness decreased substantially under all test conditions, accompanied by an improvement in circumferential uniformity.

**Figure 12:**
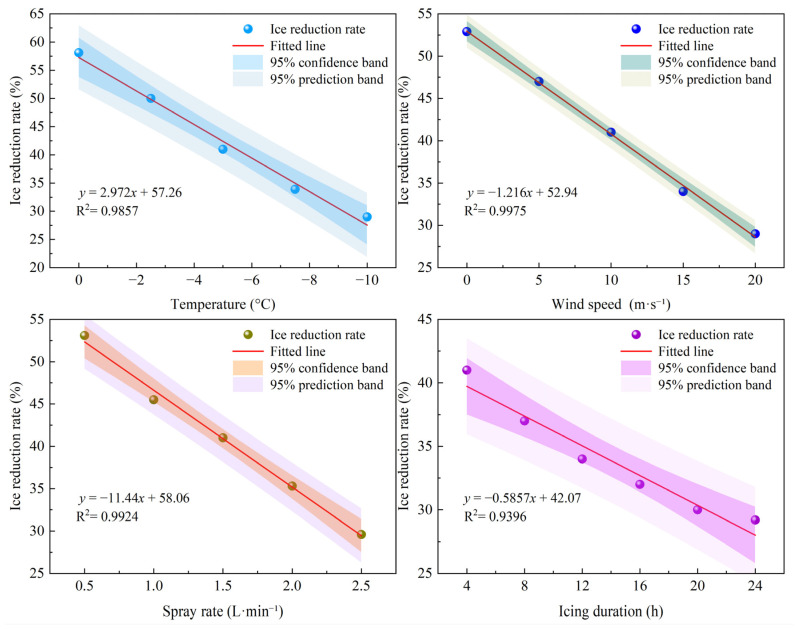
Ice reduction rate of the superhydrophobic coating on the aged circular steel tube under different test conditions.

As shown in Figure 12, the ice reduction performance of the coating depended strongly on the environmental conditions. At relatively high temperatures, low wind speeds, and low spray rates, the Cassie–Baxter composite interface remained stable, allowing droplet rebound and interfacial thermal insulation to act synergistically. At 0 °C, the ice reduction rate reached 58%, the highest value across all test conditions. As the temperature decreased and the wind speed and spray rate increased, greater droplet supercooling and higher dynamic impact pressure promoted a transition from the Cassie state to the Wenzel state, progressively weakening the core ice-reduction mechanisms. Under the most severe conditions, the ice reduction rate decreased to approximately 29%. During prolonged icing, the micro/nanoscale structures were gradually infiltrated and filled as ice accumulated, resulting in a gradual decline in the ice reduction rate. Nevertheless, the coating maintained an ice reduction rate of 29% after 24 h, indicating stable performance during long-term icing exposure.

### 4.2. New Galvanized Circular Steel Tube

**Figure 13:**
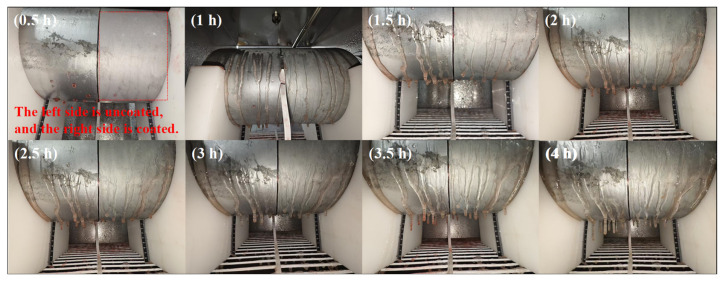
Comparison of anti-icing performance between uncoated and superhydrophobic-coated new galvanized circular steel tubes.

The new hot-dip galvanized circular steel tube had a dense and intact zinc coating, low surface roughness, and a stronger tendency for droplet slippage, representing the typical surface condition of primary members in newly constructed long-span transmission towers. Compared with the aged member, the uncoated surface exhibited a lower droplet capture efficiency. After the application of the superhydrophobic coating, the relatively smooth substrate enabled more uniform formation of the coating micro/nanostructures and improved interfacial stability. The icing characteristics under the reference condition are shown in Figure 13.

**Figure 14:**
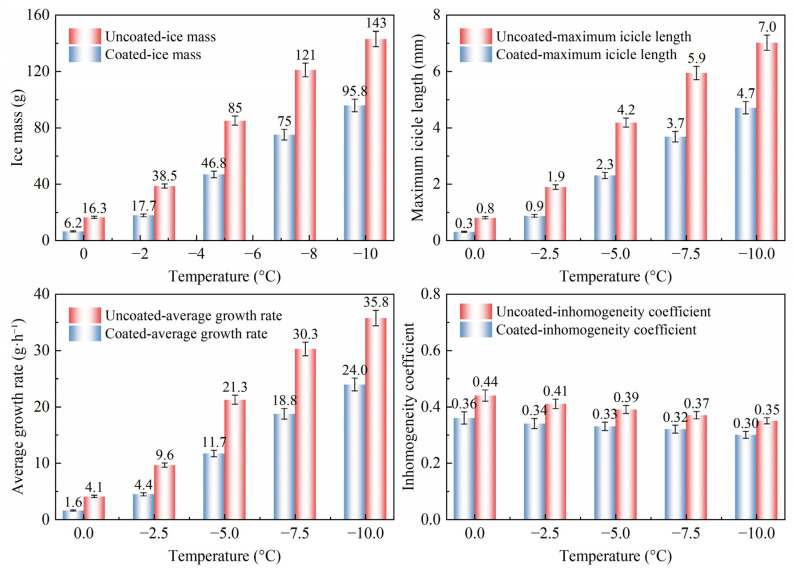
Icing characteristics of the new galvanized circular steel tube at different ambient temperatures.

**Figure 15:**
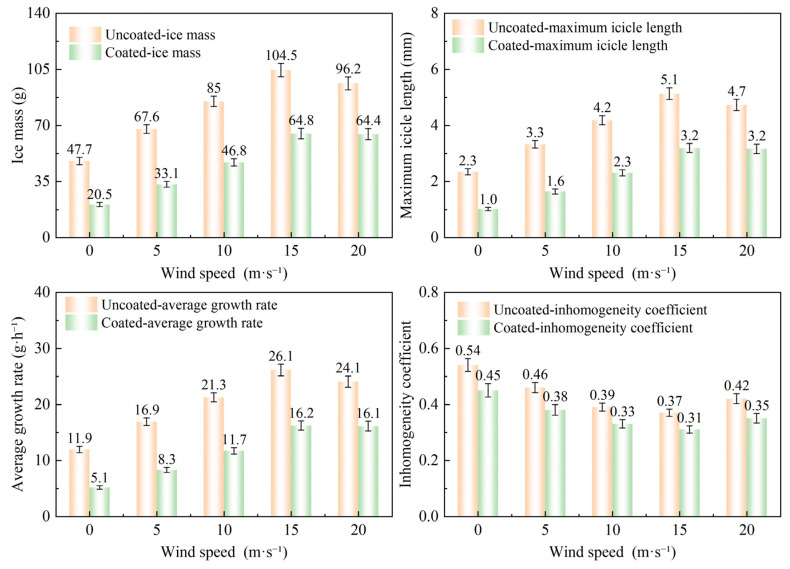
Icing characteristics of the new galvanized circular steel tube at different wind speeds.

**Figure 16:**
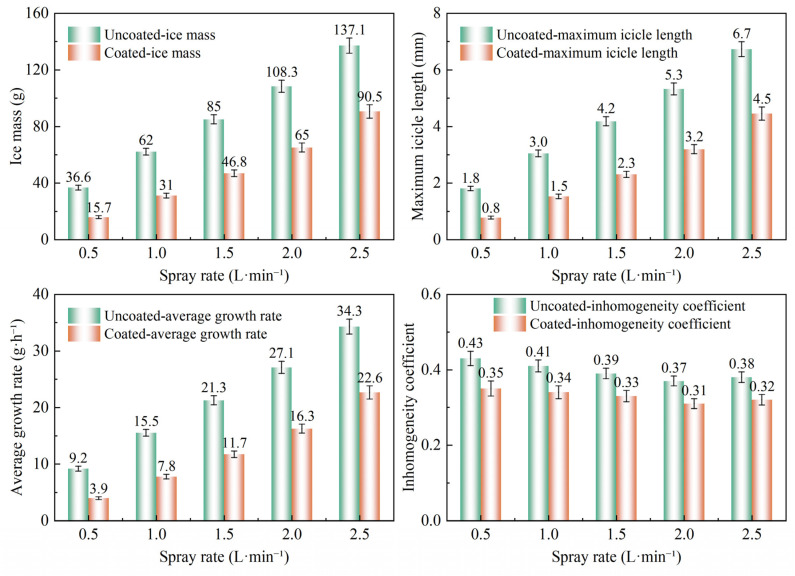
Icing characteristics of the new galvanized circular steel tube at different spray rates.

**Figure 17:**
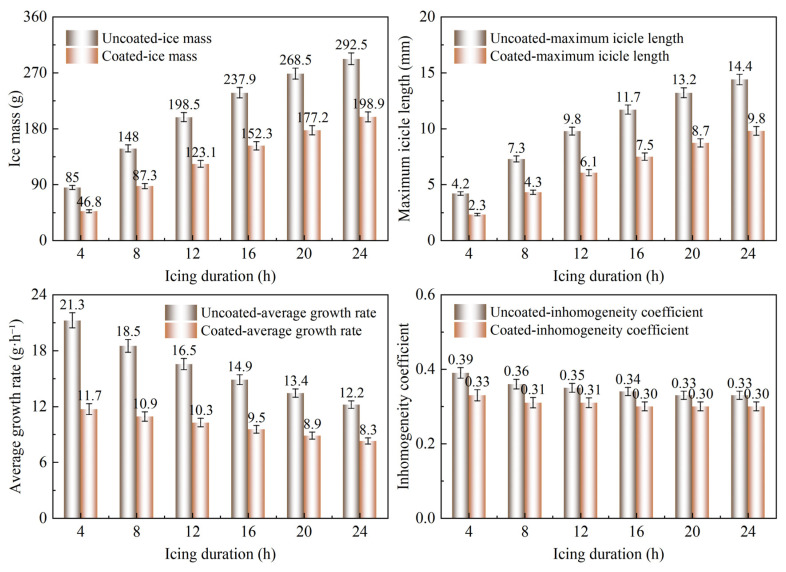
Icing characteristics of the new galvanized circular steel tube at different icing durations.

The overall variation in icing on the uncoated reference surface was similar to that observed for the aged member, although the absolute ice mass was lower. Under the reference condition, the ice mass was 12% lower than that of the aged circular steel tube. This difference was primarily attributed to the smaller droplet adhesion area on the smooth surface, which allowed discrete droplets to roll off more readily. As shown in Figure 14, the circumferential ice-thickness Inhomogeneity coefficient was generally lower than that of the aged member over the investigated temperature range because droplet spreading along the surface was more limited. As shown in Figure 15, ice mass reached its maximum at a wind speed of 15 m/s. As the ice–solid interfacial adhesion strength was lower, secondary wind-induced ice detachment was more pronounced at high wind speeds, resulting in a greater reduction in ice mass at 20 m/s. As shown in Figure 16, droplet roll-off was prominent at low spray rates, leading to relatively slow ice growth. However, once a continuous water film formed, the growth rate increased rapidly. As shown in Figure 17, ice mass continued to exhibit a decelerating growth pattern over prolonged icing durations. From 4 to 24 h, the average ice growth rate decreased by 42%, and the steady-state growth rate was slightly lower than that of the aged member.

**Figure 18:**
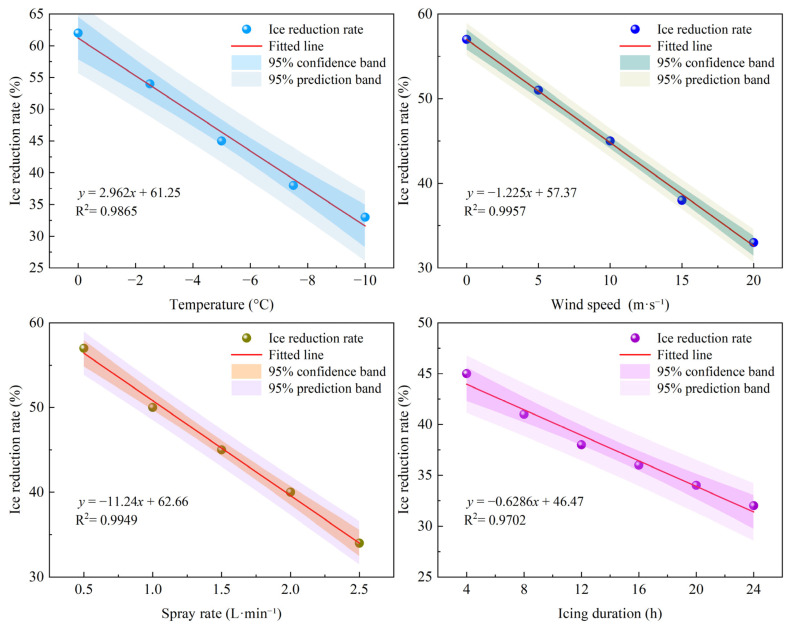
Ice reduction rate of the superhydrophobic coating on the new galvanized circular steel tube under different test conditions.

As shown in Figure 18, the superhydrophobic-coated surface exhibited higher ice reduction performance than the aged member under all test conditions. Under the reference condition, the ice reduction rate reached 45%, which was 4 percentage points higher than that of the aged circular steel tube. This performance advantage arose because the smooth substrate preserved the continuity of the coating micro/nanostructures, increased the critical wetting pressure of the Cassie–Baxter interface, and reduced the number of substrate defects that could serve as nucleation sites for droplet infiltration, thereby improving interfacial stability. The effects of the environmental parameters on the ice reduction rate followed the same general trends as those observed for the aged member, but the performance deterioration under severe conditions was less pronounced. At −10 °C and 20 m/s, the ice reduction rate remained approximately 33%. During prolonged icing, the rate decreased gradually but remained at 32% after 24 h. This reduction was smaller than that of the aged member, indicating better stability during long-term icing exposure.

### 4.3. New Galvanized Angle Steel

**Figure 19:**
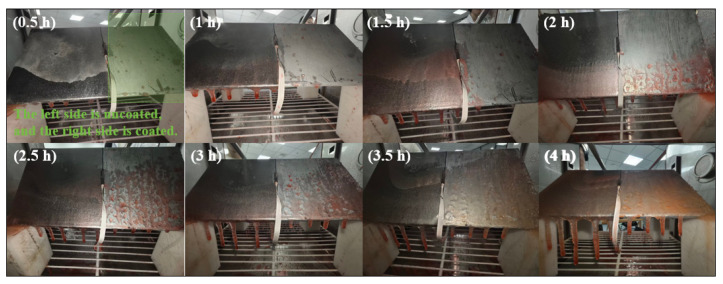
Comparison of anti-icing performance between uncoated and superhydrophobic-coated new galvanized angle steel.

Equal-leg angle steel is a representative cross-section used for diagonal and secondary members in long-span transmission towers. Its sharp-edged geometry substantially disturbs the surrounding flow field, resulting in higher droplet collision efficiency than that of circular members. Local icicles also tend to form at the edges, producing a more pronounced nonuniform circumferential ice distribution. The specimens had new hot-dip galvanized surfaces with dense and intact zinc coatings. Their icing characteristics under the reference condition are shown in Figure 19.

**Figure 20:**
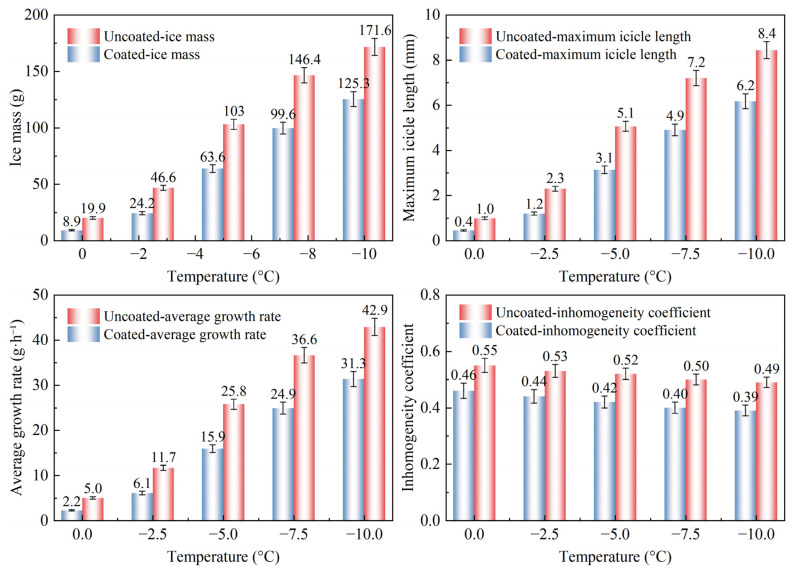
Icing characteristics of the new galvanized angle steel at different ambient temperatures.

**Figure 21:**
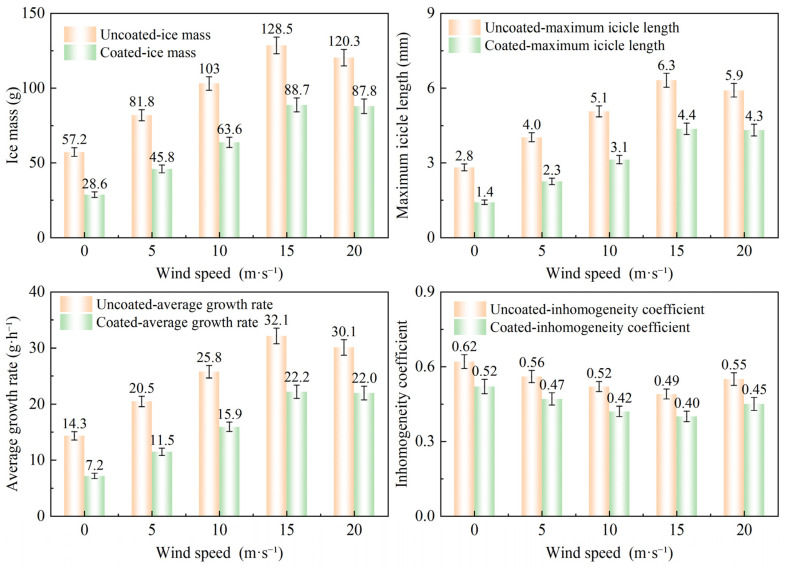
Icing characteristics of the new galvanized angle steel at different wind speeds.

**Figure 22:**
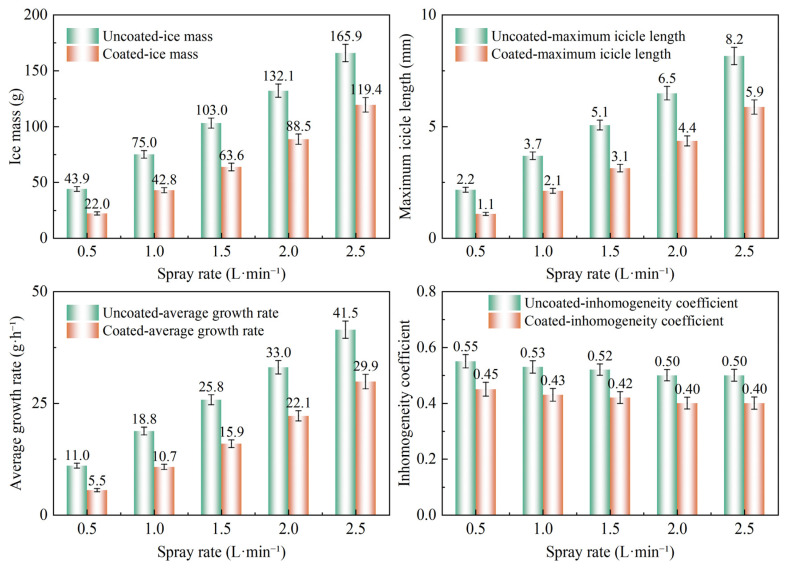
Icing characteristics of the new galvanized angle steel at different spray rates.

**Figure 23:**
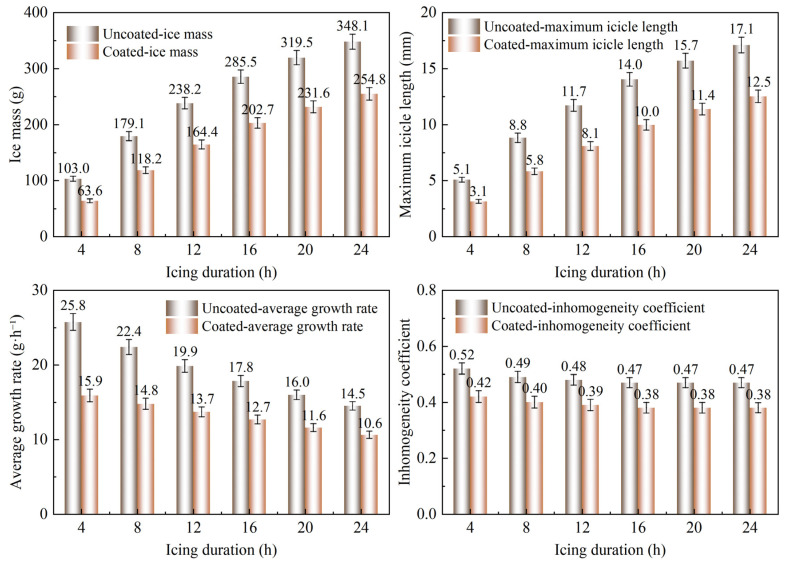
Icing characteristics of the new galvanized angle steel at different icing durations.

The overall variation in icing on the uncoated reference surface was similar to that observed for the circular steel tubes, although both the absolute ice mass and spatial nonuniformity were markedly greater. Under the reference condition, the ice mass was 21% higher than that of the new galvanized circular steel tube. This difference was primarily attributed to local flow distortion near the edges, which substantially increased the local collision efficiency. The Inhomogeneity coefficient reached 0.62, exceeding that of the circular members and reflecting the pronounced formation of edge icicles. As shown in Figure 20, ice mass increased monotonically with a higher degree of supercooling. At a higher degree of supercooling, icicles grew more rapidly along the edges, and the Inhomogeneity coefficient remained high throughout the investigated temperature range. As shown in Figure 21, ice mass initially increased and then decreased with increasing wind speed, reaching its maximum at 15 m/s. The sharp-edged geometry enhanced droplet capture, resulting in faster ice growth than on the circular tubes at low wind speeds. At high wind speeds, however, edge-induced droplet splashing became more pronounced, leading to a greater reduction in ice mass at 20 m/s. As shown in Figure 22, droplet roll-off was prominent at low spray rates, resulting in relatively slow ice growth. Once a continuous water film formed, the growth rate increased rapidly, and the ice mass of the uncoated specimen increased by 278% over the investigated spray-rate range. As shown in Figure 23, ice mass continued to exhibit a decelerating growth pattern over prolonged icing durations. From 4 to 24 h, the average ice growth rate decreased by 42%. As the edge icicles reached a steady growth state earlier, the overall reduction in growth rate was slightly smaller than that observed for the circular steel tubes.

For the new galvanized angle steel, the calculated inhomogeneity coefficient was interpreted together with the observed ice morphology. The sharp-edged geometry promoted local droplet collection and icicle growth near the windward edges, which may not be fully captured by a limited number of thickness measurements. Therefore, the larger inhomogeneity coefficient of the angle steel should be understood as a comparative indicator of stronger spatial nonuniformity, supported by both the representative thickness measurements and the recorded macroscopic morphology.

**Figure 24:**
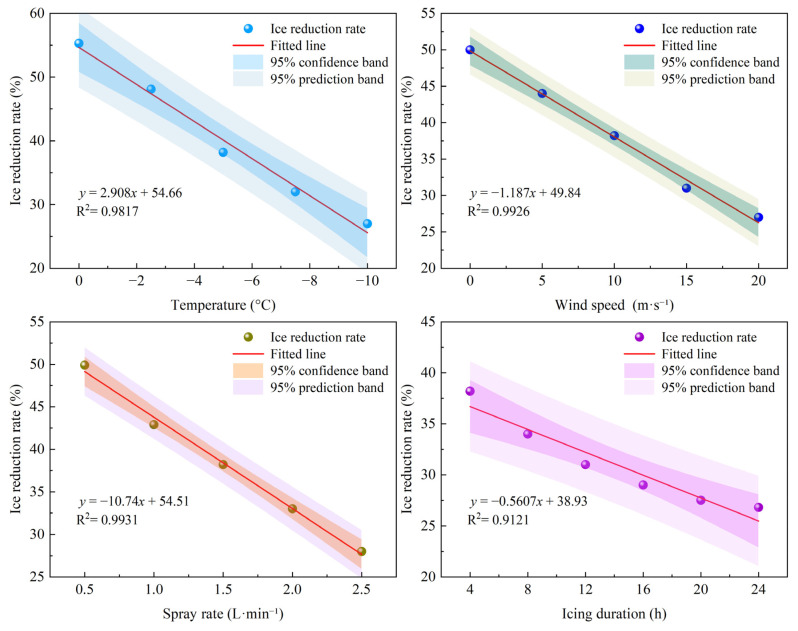
Ice reduction rate of the superhydrophobic coating on the new galvanized angle steel under different test conditions.

As shown in Figure 24, the application of the superhydrophobic coating markedly reduced both ice mass and the inhomogeneity coefficient under all test conditions. However, its overall ice reduction performance was lower than that achieved on the new galvanized circular steel tube. Under the reference condition, the ice reduction rate was 38%. This lower effectiveness was primarily attributed to the poorer uniformity of the coating film formed at the edges, where defects were more likely to develop in the micro/nanostructures. In addition, the higher normal dynamic impact pressure of droplets at the edges more readily exceeded the critical wetting pressure of the Cassie–Baxter composite interface, resulting in localized wetting failure. The environmental parameters affected the ice reduction rate in a manner similar to that observed for the circular steel tubes, although the performance deterioration caused by increasing wind speed was more pronounced. During prolonged icing, the microstructures near the edges were infiltrated and filled by ice earlier than those on the remaining surface, causing the ice reduction rate to decline gradually. The rate remained at 27% after 24 h, indicating lower stability during long-term icing exposure than that of the circular members.

### 4.4. Multifactor Analysis Before and After Coating Application

**Figure 25:**
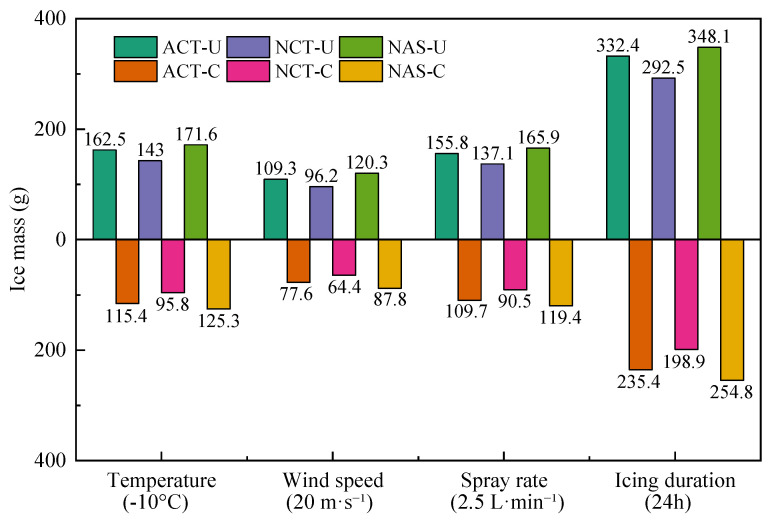
Comprehensive comparison of ice mass among all tested variants under representative environmental-variable conditions. ACT, NCT, and NAS denote aged circular steel tube, new galvanized circular steel tube, and new galvanized angle steel, respectively; U and C denote uncoated and superhydrophobic-coated specimens, respectively.

To provide an integrated comparison among all tested variants, Figure 25 summarizes the ice mass of the aged circular steel tube, new galvanized circular steel tube, and new galvanized angle steel under representative environmental-variable conditions. Both uncoated and superhydrophobic-coated specimens are included. Among the evaluation indicators used in this study, ice mass was selected for the integrated comparison because it directly represents the accumulated icing load on steel members and provides the most direct measure of coating-induced ice reduction. As shown in Figure 25, the superhydrophobic coating reduced ice mass for all three member types under the selected low-temperature, high-wind-speed, high-spray-rate, and long-duration conditions. The comparison also confirms the influence of member geometry and surface state: the new galvanized circular steel tube generally exhibited the lowest ice mass and the strongest coating response, whereas the new galvanized angle steel showed greater ice accumulation because its sharp-edged geometry promoted local droplet collection and edge icing.

For the uncoated specimens, the effects of the environmental variables followed the physical mechanisms described in Section 2. Ambient temperature mainly controlled the freezing efficiency through the heat-transfer driving force; therefore, ice mass increased as supercooling intensified. Wind speed affected droplet inertia and collision efficiency, leading to an initial increase in ice mass followed by a decrease when splashing and secondary detachment became more pronounced at higher wind speeds. Spray rate controlled the incoming liquid water flux and therefore produced an approximately monotonic increase in ice mass. Icing duration affected the accumulation process through the gradual growth of the ice layer and the associated increase in thermal resistance, resulting in a decelerating growth pattern over time. These trends were consistent among the three member types, although the absolute ice mass differed because of cross-sectional geometry and surface condition.

After coating application, the governing factors shifted from ice accumulation alone to the stability of the superhydrophobic wetting state under coupled environmental loading. Under milder conditions, droplet rebound, reduced solid–liquid contact, and interfacial thermal resistance remained effective, resulting in a marked decrease in ice mass. Under harsher conditions, however, the ice-reduction effect weakened because accelerated freezing shortened the droplet spreading-retraction time, high wind speed increased impact pressure, and high liquid water supply promoted continuous water-film formation. These effects increased the probability of local Cassie-to-Wenzel wetting transition and reduced the effectiveness of the coating. Prolonged icing further promoted ice accumulation within surface microstructures, which explains the gradual decline in coating performance with icing duration.

The coating response also depended strongly on the member geometry and substrate state. Smooth circular members favored more uniform coating formation and more stable droplet rebound, whereas the aged circular steel tube contained surface defects that could promote local wetting and ice nucleation. The new galvanized angle steel exhibited the weakest relative coating response because its sharp edges intensified droplet impingement, local water retention, and edge icicle growth. Therefore, the present results indicate that superhydrophobic coatings are more effective on smooth circular tower members than on sharp-edged members under severe icing conditions. The stability discussed here should be interpreted as anti-icing performance retention within the tested laboratory duration. Although the coating maintained an ice-reduction effect after 24 h of continuous icing, this result does not directly verify wear resistance, adhesion strength, abrasion resistance, or long-term outdoor aging resistance. These aspects require dedicated durability tests, including abrasion cycling, adhesion testing, freeze–thaw exposure, and post-icing surface characterization.

## 5. Conclusions

This study conducted multifactor coupled icing simulation tests on three representative steel members of long-span transmission towers in the Yangtze River basin, systematically compared the icing evolution of uncoated and superhydrophobic-coated surfaces, and clarified the coupling effects of environmental parameters, member characteristics and coating anti-icing performance. The main conclusions are drawn as follows:

(1) The icing process of uncoated members is dominated by freezing efficiency, collision efficiency and liquid water content. Ice mass increases with supercooling degree and spray rate, and first rises then decreases with wind speed. Over 4–24 h of continuous icing, the average ice growth rate drops by 42–43%, consistent with the decelerating growth pattern induced by cumulative thermal resistance of the ice layer.

(2) Cross-sectional geometry and substrate condition jointly affect the absolute ice mass and its spatial distribution. Under the reference condition, the ice mass of new galvanized angle steel is 21% higher than that of new galvanized circular steel tube, with a Inhomogeneity coefficient of 0.62, as sharp-edged geometry intensifies local droplet collision and icicle growth. The aged plain circular steel tube accumulates more ice due to higher surface roughness and substrate defects.

(3) The superhydrophobic coating reduces ice mass for all three member types, with its performance varying with member geometry and substrate state. Under the reference condition, the ice reduction rate reaches 45% for the new galvanized circular steel tube, 41% for the aged plain circular steel tube, and 38% for the new galvanized angle steel. Circular cross-sections and smooth substrates benefit the continuity of coating micro/nanostructures and the stability of the Cassie–Baxter composite interface.

(4) The ice reduction performance of the coating exhibits strong environmental dependence. Mild supercooling, low wind speed and low spray rate facilitate droplet rebound, roll-off and interfacial thermal insulation, while harsh conditions accelerate freezing, raise dynamic impact pressure and induce localized wetting, thus weakening the anti-icing effect. After 24 h of continuous icing, the ice-reduction rates remained at 27–32% for the three members, indicating retained anti-icing performance within the tested duration. Nevertheless, these findings are limited to controlled laboratory icing conditions. Direct wear, adhesion, abrasion, and post-icing surface characterization tests were not included in the present study, and the long-term mechanical durability and outdoor aging resistance of the coating require further systematic investigation.

## 6. Outlook

Although the specimens used in this study retained the original cross-sectional dimensions of full-scale engineering tower members, the icing tests were conducted on 300 mm long member segments under controlled laboratory conditions. The present results therefore provide member-level evidence for the effects of cross-sectional geometry, substrate condition, environmental loading, and superhydrophobic coating on icing behavior, but they should not be interpreted as a complete prediction of full-scale tower performance under natural service environments. Future work should focus on validating the transferability of these findings under coupled field conditions. In particular, full-scale or large-scale tower-member tests should be conducted under natural icing, turbulent wind, structural vibration, ultraviolet exposure, pollutant deposition, corrosion, and repeated freeze–thaw aging. Long-term outdoor exposure tests are also needed to evaluate coating adhesion, abrasion resistance, wetting-state stability, and post-icing surface degradation. Such studies will help establish durability criteria and application guidelines for superhydrophobic coatings used in passive anti-icing protection of long-span transmission towers.

## Figures and Tables

**Table 1:** Experimental variables and factor levels.

Variable Type	Condition ID	Temperature (°C)	Wind Speed (m·s^−1^)	Spray Rate (L·min^−1^)	Icing Duration (h)
Temperature	T_1_	0	10	1.5	4
	T_2_	−2.5			
	T_3_	−5			
	T_4_	−7.5			
	T_5_	−10			
Wind speed	W_1_	−5	0	1.5	4
	W_2_		5		
	W_3_		10		
	W_4_		15		
	W_5_		20		
Spray rate	S_1_	−5	10	0.5	4
	S_2_			1.0	
	S_3_			1.5	
	S_4_			2.0	
	S_5_			2.5	
Icing duration	D_1_	−5	10	1.5	4
	D_2_				8
	D_3_				12
	D_4_				16
	D_5_				20
	D_6_				24

## Data Availability

The original contributions presented in this study are included in the article. Further inquiries can be directed to the corresponding author.
